# New Method of Avoiding Underestimation of Caries Incidence and Its Association with Possible Risk Factors in Japanese University Students: A Prospective Cohort Study

**DOI:** 10.3390/ijerph19042490

**Published:** 2022-02-21

**Authors:** Daisuke Ekuni, Naoki Toyama, Yoshiaki Iwasaki, Manabu Morita

**Affiliations:** 1 Department of Preventive Dentistry, Graduate School of Medicine, Dentistry and Pharmaceutical Sciences, Okayama University, 2-5-1, Shikata-cho, Kita-ku, Okayama 700-8558, Japan; pu171qxi@s.okayama-u.ac.jp (N.T.); mmorita@md.okayama-u.ac.jp (M.M.); 2 Health Service Center, Okayama University, 2-1-1, Tsushima-naka, Kita-ku, Okayama 700-8530, Japan; yiwasaki@okayama-u.ac.jp

**Keywords:** dental caries, risk factors, disease progression, cohort studies

## Abstract

The objective of this three-year prospective cohort study was to investigate the association between a new definition of an increase in dental caries and risk factors in Japanese young adults. Data of Okayama University students who volunteered to undergo oral examinations and answer questionnaires in 2015 and 2018 were analyzed. The status of filled teeth and the status of occlusal/proximal surfaces of filled or decayed teeth were recorded. An increase in dental caries was defined as a change in the status of filled teeth and/or an increase in dental caries of occlusal and proximal surfaces. A total of 393 participants (18.2 ± 0.8 years) were analyzed. First and second molars showed a high prevalence of dental caries. Of the participants, 144 (36.6%) showed an increase in dental caries. In all the participants and in the females, the decayed, missing, and filled teeth (DMFT) score and history of orthodontic treatment at baseline were significantly associated with an increase in dental caries (*p* < 0.05) in logistic regression analyses. In the males, the DMFT score and the daily frequency of snacking (≥2) at baseline were significantly associated with an increase in dental caries (*p* = 0.04). The DMFT score and history of orthodontic treatment at baseline can be risk factors for an increase in dental caries using the new definition in young adults.

## 1. Introduction

Dental caries is one of the most prevalent oral diseases worldwide [1]. It has negative impacts on oral health and quality of life [2] and is one of the main reasons for the extraction of permanent teeth in Japan [3]. Dental caries is a multifactorial disease involving interactions between the diet, the host, and the microorganisms over time [4], as well as other risk factors [5,6,7,8,9].

Although global evidence shows that the prevalence of dental caries has been decreasing over the past four decades, this trend is mainly observed in high-income countries, and its decline is seen in 12-year-old children [10,11]. According to the national data in Japan, the dental caries prevalence among school children has decreased over the last 30 years [12]. The decayed, missing, and filled teeth (DMFT) score reached 0.70 for 12-year-old children in 2020 [12]. However, dental caries prevalence is still high in young adults and increases from 31.76% at 12 years of age to 47.95% at 17 years of age [12]. Thus, dental caries remains a major problem and should be a focus in the promotion of oral health in young adults.

The index used most commonly for dental caries is the DMFT score [13]. However, the cohort studies using the DMFT system in epidemiological surveys have underestimated the history of dental caries because refilled dental materials and the increased number of decayed surfaces due to recurrent dental caries are not recorded. There are some cohort studies of young adults using the decayed, missing, and filled surfaces (DMFS) score that investigates the surfaces of each tooth [14,15,16,17,18]. However, this also cannot account for refilled dental materials. In fact, unlike in clinical studies, it is difficult to investigate refilled dental materials in epidemiological surveys without dental charts and X-ray scans. Thus, new definitions are required to avoid underestimation of dental caries incidence.

In order to avoid underestimation of dental caries incidence, we focused on the change in dental restoration, for example, from resin filling to metal inlay, and the increase in the number of dental surfaces with a history of caries in each tooth. There have been few epidemiological cohort studies that monitored such changes in dental caries history. In young adults, dental caries is mostly prevalent in occlusal surfaces, followed by proximal surfaces [19]. Therefore, the aim of this prospective cohort study was to investigate the association of caries incidence determined using this new approach and risk factors in Japanese young adults. We hypothesized that using this new approach to monitor changes in dental caries would avoid underestimation of dental caries incidence and allow the identification of previously unknown risk factors.

## 2. Materials and Methods

### 2.1. Study Design and Setting

This was a 3-year prospective cohort study. The baseline recruitment was performed in April 2015. Re-examination at follow-up was performed 3 years after the baseline examination (April 2018). The study location was the Okayama University Health Service Center at Okayama University of Japan.

### 2.2. Participants

The study participants were recruited from the first-year students of Okayama University in 2015. These students were undergraduate students from all faculties who volunteered to undergo an oral examination and answer questionnaires at the Okayama University Health Service Center in April 2015. Some of them then volunteered to undergo an oral examination and answer questionnaires during the follow-up survey in April 2018. Included were the students who agreed to participate in the study and for whom complete data of both the oral examination and the questionnaires were available. Excluded were the students who did not undergo the oral examination or for whom only incomplete data were available. 

### 2.3. Ethics Procedures and Informed Consent 

The study protocol was approved by the Ethics Committee of the Okayama University Graduate School of Medicine, Dentistry, and Pharmaceutical Sciences and the Okayama University Hospital (No. 1060). All the participants provided verbal informed consent for participation in this study. All the methods were performed in accordance with the relevant guidelines and regulations. The present study was performed in accordance with the STROBE Statement.

### 2.4. Oral Examination

Six trained dentists (T.A., S.M., K.K., M.Y.-T., A.T.-T. and D.E.) performed oral examinations. The number of teeth present including third molars was recorded at baseline and follow-up. The World Health Organization (WHO) diagnostic criteria for history of dental caries [13] were used to evaluate the DMFT score. Furthermore, the status of filled teeth (amalgam filling/resin filling/metal inlay/full-coverage crown) and of occlusal (foramen cecum/incisal in case of anterior teeth) and proximal surfaces of filled or decayed teeth was recorded at baseline and follow-up. 

A modified version of the Index of Orthodontic Treatment Need [20] was used to evaluate malocclusion at baseline. The oral hygiene status was evaluated by visually inspecting selected teeth based on the Debris Index-Simplified (DI-S) [21]. The dentists repeatedly practiced the dental caries and malocclusion evaluations in two volunteers for two weeks. For the oral examination, intra- and inter-agreements were good (Kappa statistic > 0.8).

### 2.5. Questionnaire

Self-reported questionnaires on risk factors for dental caries were used at baseline based on the previous reports [22,23,24], including age, sex (male/female), use of fluoride toothpaste (yes/no), knowledge of the effectiveness of fluoride (yes/no), history of topical application of fluoride in elementary, junior high, and high schools (yes/discontinued/never), daily frequency of tooth brushing (≥2/<2), daily frequency of snacking (≥2/<2), smoking status (current/past/never), use of a dental floss (yes/no), history of orthodontic treatment (yes/no), regular dental checkups (yes/no), and dry mouth (yes/no). Since the WHO recommends twice-daily tooth brushing with a fluoride toothpaste to prevent dental caries, the two questionnaires were combined, and “daily frequency of tooth brushing with a fluoride toothpaste” (≥2/<2) was used.

### 2.6. Main Outcome

The main outcome of this study was an increase in dental caries. An increase in dental caries was defined as an increase in the DMFT score and/or changes in the status of filled teeth, and/or an increase in dental caries of occlusal and proximal surfaces during the 3-year study period. The participants were then classified into the increase and no-increase groups.

### 2.7. Bias

The dentists encouraged the patients to undergo re-examinations to avoid dropouts (attrition bias). Calibrated methods were used to minimize information bias. All cases without incomplete data were used, and students with and without follow-up were compared to check for selection bias.

### 2.8. Sample Size Estimation

Since there were no previous reports that investigated the association between an increase in dental caries using our definitions and risk factors, sample size estimation was not performed.

### 2.9. Statistical Analysis

The Mann–Whitney *U* test, chi-squared test, or Fisher’s exact test was used to assess significant differences in variables between the two groups. The Wilcoxon signed-rank test or McNemar’s test was used to assess significant differences in variables between the baseline and the follow-up. The associations between an increase in dental caries and all the independent variables that are risk factors for dental caries were analyzed by means of logistic regression. Subgroup analyses by sex were also performed. All the missing data were excluded in this study. All the analyses were performed using SPSS 25.0 J for Windows (IBM Japan, Tokyo, Japan). Values of *p* < 0.05 were considered significant.

## 3. Results

**Figure 1:**
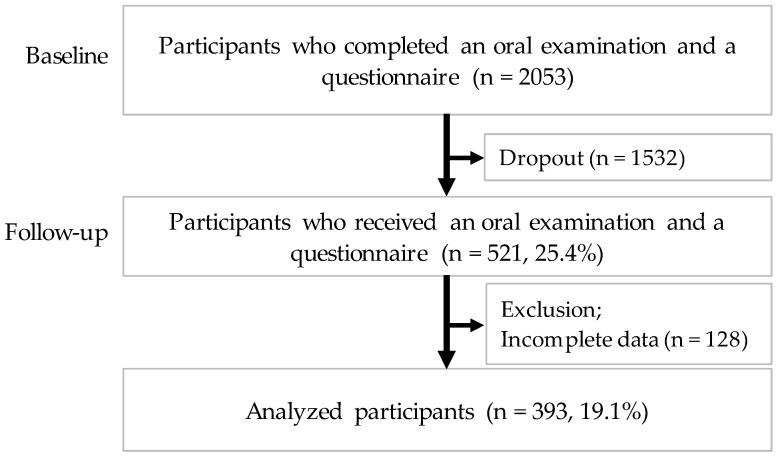
Flowchart of the study.

Figure 1 shows the study flowchart. Of the 2053 participants who completed the oral examination and the questionnaire, 393 participants were analyzed after 3 years (follow-up rate: 19.1%). There were no significant differences in the related factors at baseline between the participants with follow-up and the students without follow-up.

Table 1 shows the characteristics of the participants at baseline. Overall, the mean age at baseline (± the standard deviation (SD)) of the analyzed participants was 18.2 ± 0.8 (median, 18.0; range, 18–29) years. The mean DMFT score at baseline (± the standard deviation) was 1.6 ± 2.8 (median, 0.0; range, 0.0–22.0). The number (%) of dental caries-free participants (DMFT = 0) at baseline was 220 (56.0%). There were no participants with dry mouth.

The mean DMFT score at follow-up (±SD) was 2.5 ± 3.7 (median, 1.0; range, 0.0–22.0). It increased significantly (Wilcoxon signed-rank test, *p* < 0.001), and the number of dental caries-free participants decreased significantly during the 3-year period (from 220 to 176; McNemar’s test, *p* < 0.001).

Table 2 shows the tooth type and changes in dental caries. The highest prevalence of decayed teeth was the upper second molar at baseline and the upper third molar at follow-up. Missing teeth were only observed in the third molar at follow-up. The highest prevalence of filled teeth was the lower first molar at baseline and follow-up. The highest increase in dental caries was observed in the upper second molar during the 3-year period (72 teeth). The major status of filled teeth was resin. The major surface of filled teeth was the occlusal surface. The highest prevalence of dental caries in the occlusal surface was the lower first molar at baseline and follow-up.

Table 3 shows the differences in baseline factors between the increase and no-increase groups. According to the new definition of an increase in caries, 144 (36.6%) of the 393 students showed an increase in dental caries. Of them, nine (2.3%) students were underestimated compared to the cases with an increase in the DMFT score. The DMFT score at baseline was significantly higher in the increase group than in the no-increase group among all the participants, males, and females (all, *p* < 0.001). The prevalence of the history of orthodontic treatment was significantly higher at baseline in the increase group than in the no-increase group among all the participants (*p* = 0.004) and the females (*p* = 0.002). In the males, the prevalence of a high frequency of snacking (≥2/day) was significantly higher in the increase group than in the no-increase group (*p* = 0.021).

Table 4 shows the results of logistic regression analyses. In all the participants and the females, the DMFT score and the history of orthodontic treatment at baseline were significantly associated with an increase in dental caries (both *p* < 0.05). In the males, the DMFT score and the daily frequency of snacking (≥2) were significantly associated with an increase in dental caries (both *p* < 0.05). 

## 4. Discussion

This 3-year cohort study showed that the increase in dental caries using the new definition was significantly associated with the DMFT score and the history of orthodontic treatment at baseline among all the participants and the females. On the other hand, in the males, the increase in dental caries was significantly associated with the DMFT score and the daily frequency of snacking at baseline. To the best of our knowledge, this is the first study to show results that were confirmed using this new definition. The DMFT score, the history of orthodontic treatment, and the daily frequency of snacking at baseline may be risk factors for an increase in dental caries.

The history of dental caries (DMFT > 0) at baseline was a risk factor for an increase in dental caries in university students aged 18–29 years. Previous cohort studies reported that a history of caries was a risk factor for dental caries incidence in people aged 2–30 years [17,25,26,27,28]. People with a history of dental caries (DMFT > 0) are usually classified as a risk group [26]. These findings support the present results. On the other hand, the findings in the present study suggest that it is important to prevent dental caries in students before they enter university. Dentists and healthcare providers should continue to provide and also expand public health methodology for dental caries prevention, including supervised regular use of fluoride mouth rinses [29] and water fluoridation [30].

In the males, a high frequency of snacking was a risk factor for an increase in dental caries. A cariogenic diet, including sugar, is an important risk factor for dental caries [31]. Sugar restriction (amount and frequency) is important for dental caries prevention [6]. Thus, especially for males, control of snacking may be an important strategy to implement for preventing an increase in dental caries during university life.

In the females, a history of orthodontic treatment was a risk factor for an increase in dental caries. The abundance of *Streptococcus mutans* increases during and after orthodontic treatment [24,32]. The number of females with a history of orthodontic treatment was significantly higher than that of males in the present study (43 vs. 32, *p* < 0.038). Thus, only in females a history of orthodontic treatment may be related to an increase in dental caries.

Application of fluoride (history of topical application of fluoride in elementary, junior high, and high school and daily frequency of tooth brushing with a fluoride toothpaste) as well as knowledge of the effectiveness of fluoride was not associated with an increase in dental caries in university students. This may be due to the small number of students who use a fluoride toothpaste and have knowledge of the protective effects of fluoride despite the fact that the recent market share of fluoridated toothpastes in Japan was reported to be about 90% [33]. 

A review has suggested that when fluoride is appropriately used, the association between sugar consumption and dental caries is very low or absent [6]. An FDI World Dental Federation task group emphasized that dental caries should be considered a behavioral disease with a bacterial component and that it needs to be prevented [34]. Based on that recommendation, dentists should put an increased effort toward encouraging students to brush their teeth with a fluoridated toothpaste twice daily, as well as to reduce the frequency of sugar intake.

Unfortunately, the new definition of an increase in dental caries has limited utility. The hope was that the new definition would improve on a limitation of the DMFT/DMFS score and reduce the underestimation of the increase in caries in epidemiological studies without dental charts or X-ray scans. However, the difference between the new definition and the DMFT score was not large. That is, with the new definition, only nine additional participants (2.3%) were detected compared to the original definition (increase in the DMFT score). Furthermore, the results of the logistic regression analysis using the case of an increase in the DMFT score were comparable to the present data (data not shown); i.e., the increase in the DMFT score was significantly associated with the DMFT score and the history of orthodontic treatment at baseline.

The new definition that evaluates the increase in dental caries may have some potential. Since middle-aged and elderly persons tend to receive treatment for dental caries or retreatment, changes in dental materials and increases in the number of surfaces with dental caries are more obvious than in young adults [35]. Even if a tooth with composite resin is replaced by a metal inlay due to recurrent caries, according to the original definition (DMFT score), there is no increase in dental caries. Thus, the new definition could be useful for avoiding underestimation of changes in dental caries among middle-aged and elderly persons in epidemiological surveys.

The highest increase in dental caries was observed in the upper second molar during the 3-year period (72 teeth) in this study. Dental caries of occlusal surfaces was the most prevalent. The highest prevalence of dental caries of occlusal surfaces was in the lower first molar at baseline and at follow-up. First and second molars overall have a high risk for dental caries, and the prevalence of dental caries in occlusal surfaces is higher than that in proximal surfaces [36,37,38,39,40]. The trend in the present study was supported by these previous reports.

In the present study, the mean DMFT scores (±SD) at baseline and follow-up were 1.6 ± 2.8 and 2.5 ± 3.7, respectively, in the university students aged 18–29 years. Although the sample size and period in the present study differed from those in previous studies comprising young adults aged 18–24 years [22,41,42], the mean DMFT scores were within the same range. However, the mean DMFT scores in this study were lower than those reported in a Japanese national survey of dental diseases in 2016 (3.1 for those aged 15–24 years) [35].

The present study had some limitations. Firstly, the follow-up rate was low because undergoing an oral examination was voluntary. Although the students were encouraged to undergo the examination, many students did not participate. Thus, a selection bias may have been present, and it could lead to an overestimation or underestimation of the true relationship. However, there were no significant differences in the related factors at baseline between the participants with follow-up and the students without follow-up. Thus, the effects on the results might have been small. Secondly, possible confounders such as biological factors, socioeconomic factors [23], amount of the remaining tooth structure, or presence of the post and the core [43,44,45] were not considered in the present study. Future studies need to assess the effects of these factors. Third, all the participants were recruited from among students at Okayama University, which may limit the ability to extrapolate these findings to the general population of young people. Fourth, dental charts and X-ray scans were not used. Thus, the new definition could not distinguish the increase in dental caries if the refilled materials and the surfaces were the same. Fifth, more sensitive methods such as the ICDAS (International Caries Detection and Assessment System [46]) should have been used to detect dental caries. However, the ICDAS could not be used because cleaning the teeth and drying out the surfaces are required before the examination for that system. Regarding caries risk assessment, more standardized procedures, such as CARIOGRAM or the CAMBRA system, could not be used because clinical assessments are included [47]. Use of these systems should be considered in a future study. Finally, changes in the confounding variables over the three-year study period, which may have been a potential source of bias, could not be investigated.

## 5. Conclusions

This 3-year cohort study showed that the increase in dental caries using the new definition was significantly associated with the DMFT score and the history of orthodontic treatment at baseline among all the participants and the females. On the other hand, in the males, the increase in dental caries was significantly associated with the DMFT score and the daily frequency of snacking at baseline. It is important to prevent dental caries in students. For the early stages of life, dentists and healthcare providers should continue to both provide and expand the public health methodology for dental caries prevention.

## Figures and Tables

**Table 1:** Characteristics of the participants at baseline (*n* = 393).

Variable		*n* (%)/Mean ± SD
Sex	Male/female	210/183 (53.4/46.6)
Age (y)		18.2 ± 0.8
Number of teeth present		28.4 ± 1.5
DMFT score		1.6 ± 2.8
Caries-free (DMFT = 0)	Yes	220 (56.0)
DI-S		0.4 ± 0.4
Malocclusion	Yes	139 (35.4)
Use of a fluoride toothpaste	Yes	172 (43.8)
Knowledge of the effectiveness of fluoride	Yes	110 (28.0)
History of topical application of fluoride in elementary, junior high, and high schools	Yes	83 (21.1)
	Discontinued	174 (44.3)
	Never	136 (34.6)
Daily frequency of tooth brushing	<2	43 (10.9)
Daily frequency of tooth brushing with a fluoride toothpaste	<2	191 (48.6)
Daily frequency of snacking	≥2	89 (22.6)
Smoking	Never	391 (99.5)
	Past	1 (0.3)
	Current	1 (0.3)
Use of a dental floss	No	339 (86.3)
History of orthodontic treatment	Yes	75 (19.1)
Regular dental checkups	No	289 (73.5)
Awareness of dry mouth	Yes	0 (0.0)

SD, standard deviation; DMFT, decayed, missing, and filled teeth; DI-S, Debris Score.

**Table 2:** Tooth type and changes in the history of dental caries.

Tooth Type	Number of Teeth Present	Number of Decayed Teeth	Number of Missing Teeth	Number of Filled Teeth and Status (Amalgam Filling/Resin Filling/Metal Inlay/Full-Coverage Crown)	Surfaces of Filled Teeth: Occlusal/Proximal/ Occlusal + Proximal
Upper at baseline					
8	112	1	0	4 (0/4/0/0)	4/0/0
7	780	14	0	62 (0/48/13/1)	45/16/1
6	786	7	0	104 (1/72/28/3)	79/22/3
5	776	2	0	24 (0/7/17/0)	4/20/0
4	758	2	0	15 (0/4/11/0)	4/11/0
3	780	0	0	4 (0/4/0/0)	1/3/0
2	777	0	0	20 (0/20/0/0)	5/15/0
1	786	5	0	30 (0/28/0/2)	6/22/2
Lower at baseline					
8	140	1	0	0 (0/0/0/0)	0/0/0
7	785	3	0	126 (0/114/11/1)	111/14/1
6	786	8	0	181 (2/151/26/2)	162/17/2
5	781	2	0	14 (0/6/7/1)	5/8/1
4	775	1	0	4 (0/3/1/0)	3/1/0
3	775	0	0	1 (0/1/0/0)	0/1/0
2	768	0	0	0 (0/0/0/0)	0/0/0
1	786	0	0	0 (0/0/0/0)	0/0/0
Upper at follow-up					
8	263	8	5	3 (0/3/0/0)	3/0/0
7	784	9	0	134 (0/109/21/4)	100/30/4
6	786	0	0	164 (1/122/31/10)	118/36/10
5	776	2	0	43 (0/16/23/4)	8/31/4
4	758	0	0	33 (0/19/14/0)	10/23/0
3	780	0	0	7 (0/7/0/0)	2/5/0
2	777	2	0	31 (0/31/0/0)	7/24/0
1	786	6	0	61 (0/55/1/5)	8/48/5
Lower at follow-up					
8	263	2	4	5 (0/5/0/0)	5/0/0
7	786	4	0	195 (0/174/14/7)	165/23/7
6	786	3	0	242 (0/205/31/6)	206/30/6
5	769	1	0	28 (0/17/9/2)	13/13/2
4	768	1	0	9 (0/6/3/0)	6/3/0
3	775	0	0	2 (0/2/0/0)	1/1/0
2	766	0	0	0 (0/0/0/0)	0/0/0
1	786	0	0	1 (0/0/0/1)	0/0/1

**Table 3:** Differences in baseline factors between the increase and no-increase groups.

Total				
Variable		Increase (*n* = 144)	No Increase (*n* = 249)	*p*-Value ^†^
Sex	Male	75 (52.1) *	135 (54.2)	0.683
Age (year)		18.3 ± 1.0	18.2 ± 0.6	0.721
Number of teeth present		28.3 ± 1.4	28.5 ± 1.5	0.292
DMFT score		2.9 ± 3.6	0.9 ± 1.9	<0.001
DI-S		0.4 ± 0.4	0.4 ± 0.4	0.822
Malocclusion	Yes	50 (34.7)	89 (35.7)	0.913
Knowledge of the effectiveness of fluoride	No	38 (26.4)	72 (28.9)	0.338
History of topical application of fluoride in elementary, junior high, and high schools	Yes	36 (25.0)	47 (18.9)	0.332
	Discontinued	59 (41.0)	115 (46.2)	
	Never	49 (34.0)	87 (34.9)	
Daily frequency of tooth brushing with a fluoride toothpaste	<2	68 (47.2)	123 (49.4)	0.378
Daily frequency of snacking	≥2	37 (25.7)	52 (20.9)	0.272
Smoking	Never	143 (99.3)	248 (99.6)	0.315
	Past	0 (0.0)	1 (0.3)	
	Current	1 (0.3)	0 (0.0)	
Use of a dental floss	No	124 (86.1)	215 (86.3)	0.948
History of orthodontic treatment	Yes	38 (26.4)	37 (14.9)	0.004
Regular dental checkups	No	105 (72.9)	184 (73.9)	0.832
**Males**		**Increase** **(*n* = 75)**	**No increase** **(*n* = 135)**	***p*-Value**
Age (year)		18.4 ± 1.4	18.3 ± 0.7	0.793
Number of teeth present		28.4 ± 1.6	28.6 ± 1.5	0.660
DMFT score		2.6 ± 3.3	0.9 ± 2.2	<0.001
DI-S		0.5 ± 0.4	0.4 ± 0.4	0.568
Malocclusion	Yes	26 (34.7)	44 (32.6)	0.760
Knowledge of the effectiveness of fluoride	No	27 (36.0)	45 (33.3)	0.696
History of topical application of fluoride in elementary, junior high, and high schools	Yes	18 (24.0)	24 (17.8)	0.540
	Discontinued	26 (34.7)	53 (39.3)	
	Never	31 (41.3)	58 (43.0)	
Daily frequency of tooth brushing with a fluoride toothpaste	<2	35 (46.7)	62 (54.9)	0.918
Daily frequency of snacking	≥2	22 (29.3)	22 (16.3)	0.021
Smoking	Never	74 (98.7)	134 (99.3)	0.308
	Past	1 (1.3)	0 (0.0)	
	Current	0 (0.0)	1 (0.7)	
Use of a dental floss	No	70 (93.3)	117 (86.7)	0.103
History of orthodontic treatment	Yes	13 (17.3)	19 (14.1)	0.529
Regular dental checkups	No	53 (70.7)	101 (74.8)	0.515
**Females**		**Increase** **(*n* = 69)**	**No increase** **(*n* = 114)**	***p*-Value**
Age (year)		18.1 ± 0.4	18.2 ± 0.4	0.398
Number of teeth present		28.2 ± 1.3	28.4 ± 1.5	0.307
DMFT score		2.8 ± 3.9	0.8 ± 1.6	<0.001
DI-S		0.3 ± 0.3	0.3 ± 0.3	0.878
Malocclusion	Yes	24 (34.8)	45 (39.5)	0.318
Knowledge of the effectiveness of fluoride	No	11 (15.9)	27 (23.7)	0.143
History of topical application of fluoride in elementary, junior high, and high schools	Yes	18 (26.1)	23 (20.2)	0.596
	Discontinued	33 (47.8)	62 (54.4)	
	Never	18 (26.1)	29 (25.4)	
Daily frequency of tooth brushing with a fluoride toothpaste	<2	33 (47.8)	61 (53.5)	0.277
Daily frequency of snacking	≥2	15 (21.7)	30 (26.3)	0.304
Smoking	Never	69 (100.0)	114 (100.0)	-
	Past	0 (0.0)	0 (0.0)	
	Current	0 (0.0)	0 (0.0)	
Use of a dental floss	No	54 (78.3)	98 (86.0)	0.178
History of orthodontic treatment	Yes	25 (36.2)	18 (15.8)	0.002
Regular dental checkups	No	52 (75.4)	83 (72.8)	0.420

* Note: *n* (%)/mean ± standard deviation; ^†^ Fisher’s exact/chi-squared/Mann–Whitney *U* tests; DMFT, decayed, missing and filled teeth; DI-S, Debris Score-Simplified.

**Table 4:** Risk factors at baseline for an increase in dental caries in logistic regression analyses.

Total (*n* = 393)			
Independent Variable		Adjusted Odds Ratio, * 95% Confidence Interval	*p*-Value
Sex	Male (reference)	1	
	Female	1.077, 0.676–1.716	0.755
Age (y)		0.993, 0.743–1.328	0.963
Number of teeth present		0.944, 0.810–1.100	0.459
DMFT score		1.345, 1.214–1.490	<0.001
DI-S		1.055, 0.565–1.970	0.866
Malocclusion	No (reference)	1	
	Yes	1.082, 0.673–1.738	0.745
Knowledge of the effectiveness of fluoride	Yes (reference)	1	
	No	0.855, 0.499–1.465	0.569
History of topical application of fluoride in elementary, junior high, and high schools	Yes (reference)	1	
	Discontinued	0.918, 0.496–1.701	0.786
	Never	0.985, 0.494–1.961	0.965
Daily frequency of tooth brushing with a fluoride toothpaste	≥2 (reference)	1	
	<2	0.875, 0.555–1.378	0.564
Daily frequency of snacking	<2 (reference)	1	
	≥2	1.196, 0.707–2.031	0.507
Use of a dental floss	Yes (reference)	1	
	No	1.135, 0.574–2.255	0.718
History of orthodontic treatment	No	1	
	Yes (reference)	1.976, 1.100–3.549	0.023
Regular dental checkups	Yes (reference)	1	
	No	1.127, 0.647–1.978	0.675
Males (*n* = 210)			
Age (y)		1.049, 0.763–1.442	0.770
Number of teeth present		0.988, 0.802–1.219	0.913
DMFT score		1.312, 1.155–1.490	<0.001
DI-S		1.127, 0.508–2.501	0.768
Malocclusion	No (reference)	1	
	Yes	1.039, 0.534–2.021	0.911
Knowledge of the effectiveness of fluoride	Yes (reference)	1	
	No	1.062, 0.537–2.100	0.864
History of topical application of fluoride in elementary, junior high, and high schools	Yes (reference)	1	
	Discontinued	0.997, 0.408–2.433	0.995
	Never	0.817, 0.329–2.033	0.664
Daily frequency of tooth brushing with a fluoride toothpaste	≥2 (reference)	1	
	<2	1.003, 0.534–1.887	0.992
Daily frequency of snacking	<2 (reference)	1	
	≥2	2.183, 1.037–4.597	0.040
Use of a dental floss	Yes (reference)	1	
	No	2.537, 0.755–8.519	0.132
History of orthodontic treatment	No	1	
	Yes (reference)	1.125, 0.467–2.711	0.793
Regular dental checkups	Yes (reference)	1	
	No	0.835, 0.395–1.766	0.637
Females (*n* = 183)			
Age (y)		0.908, 0.357–2.309	0.839
Number of teeth present		0.892, 0.699–1.137	0.355
DMFT score		1.445, 1.208–1.729	<0.001
DI-S		1.023, 0.351–2.977	0.967
Malocclusion	No (reference)	1	
	Yes	1.064, 0.518–2.183	0.867
Knowledge of the effectiveness of fluoride	Yes (reference)	1	
	No	0.447, 0.165–1.209	0.113
History of topical application of fluoride in elementary, junior high, and high schools	Yes (reference)	1	
	Discontinued	0.924, 0.363–2.349	0.868
	Never	1.716, 0.539–5.461	0.361
Daily frequency of tooth brushing with a fluoride toothpaste	≥2 (reference)	1	
	<2	0.769, 0.381–1.550	0.462
Daily frequency of snacking	<2 (reference)	1	
	≥2	0.691, 0.302–1.585	0.383
Use of a dental floss	Yes (reference)	1	
	No	0.562, 0.206–1.529	0.259
History of orthodontic treatment	No (reference)	1	
	Yes	3.329, 1.392–7.966	0.007
Regular dental checkups	Yes (reference)	1	
	No	1.886, 0.737–4.822	0.185

* Adjusted for all the listed independent variables; odds ratio (95% confidence interval); DMFT, decayed, missing, and filled teeth; DI-S, Debris Score-Simplified.

## Data Availability

All the relevant data are included in the manuscript.
